# Enhanced Anaerobic Digestion of Sewage Sludge Through the Integration of Thermal Hydrolysis and Bioelectrochemical Anaerobic Digestion

**DOI:** 10.3390/bioengineering13030311

**Published:** 2026-03-08

**Authors:** Chao-Wen Wang, Kai Ling Yu, Cheng-Tang Pan, Cheng-Yuan Hung, Liang-Shan Lee, Boris Tartakovsky

**Affiliations:** 1 Department of Mechanical and Electro-Mechanical Engineering, National Sun Yat-Sen University, Kaohsiung City 80424, Taiwan; pan@mem.nsysu.edu.tw; 2 Metal Industries Research and Development Centre, Kaohsiung City 81160, Taiwan; goliro@mail.mirdc.org.tw; 3 School of Engineering, Faculty of Engineering and Technology, Sunway University, No. 5, Jalan Universiti, Bandar Sunway 47500, Selangor Darul Ehsan, Malaysia; kailingy@sunway.edu.my; 4 Forest Water Environmental Engineering Co., Ltd., No. 668, Yuangang Rd., Nanzi Dist., Kaohsiung City 811, Taiwan; a353.b7900@msa.hinet.net; 5 National Research Council of Canada, 6100 Royalmount Ave, Montreal, QC H4P 2R2, Canada

**Keywords:** thermal hydrolysis pretreatment, bioelectrochemical anaerobic digestion, sewage sludge, methane production, organic loading rate

## Abstract

Thermal hydrolysis pretreatment (THP) increases the solubilization of sewage sludge, while bioelectrochemically assisted anaerobic digestion (BEAD) enhances the conversion of the solubilized organic matter into methane and improves reactor stability in the presence of inhibitory compounds. In this study, by mapping methane production in a BEAD reactor against the soluble organic loading rate (sOLR), determined from soluble chemical oxygen demand (sCOD) measurements, distinct operational regimes corresponding to different THP temperatures were identified. With the 120 °C pretreated feedstock, the BEAD reactor operated in a hydrolysis-limited regime, where increasing sOLR increased methane production but reduced conversion efficiency. Accordingly, at an sOLR of 4.5 g (L_R_ d)^−1^, a volumetric methane production rate of 0.8 L L_R_^−1^ was achieved. Increasing THP severity to 150 °C improved solids solubilization and shifted the system into a kinetically enhanced regime, in which methane production was directly proportional to sOLR, indicating improved substrate accessibility and reaction kinetics. Consequently, at an sOLR of 7.75 g (L_R_ d)^−1^, methane production reached 1.46 L L_R_^−1^. This regime-based analysis provides quantitative guidance for selecting pretreatment severity and loading strategies to maximize methane production, while maintaining stable BEAD reactor operation at high organic loads.

## 1. Introduction

With increasing urbanization and high population density in metropolitan areas, municipal wastewater treatment systems are required to handle substantially higher treatment loads within limited spatial footprints. Consequently, the generation of municipal sewage sludge has increased substantially, posing a significant challenge to urban waste management. Anaerobic digestion (AD) is widely adopted for sludge treatment due to its ability to stabilize organic matter while recovering energy as methane. However, conventional AD is limited by the recalcitrant nature of sludge solids, leading to slow hydrolysis and requiring long hydraulic retention times (HRT) and large reactor volumes [1]. Furthermore, low organic loading rates (OLR) and sluggish methane production often limit the economic feasibility of anaerobic digesters [2].

The overall AD kinetics is widely recognized to be limited by the hydrolysis of complex particulate organic materials [3]. To resolve this limitation, various pretreatment technologies have been investigated, including thermal, mechanical, and chemical approaches [4,5,6]. These strategies aim to replace slow biological hydrolysis with rapid physicochemical solubilization, thereby enabling operation at higher OLRs and shorter HRTs. Among these, the thermal hydrolysis pretreatment (THP) is recognized as an effective thermo-chemical pretreatment that can be integrated into existing infrastructures with minimal modifications [7,8]. Under hydrothermal conditions, the disruption of sludge flocs and extracellular polymeric substances (EPS) promotes the depolymerization of macromolecules into soluble intermediates. Thus, THP substantially increases soluble chemical oxygen demand (sCOD) and enhances biodegradability, substantially shortening the AD treatment time [9,10].

Pretreatment of organic solids using THP involves an important trade-off. While higher THP temperatures enhance feedstock solubilization, excessively severe hydrothermal conditions can lead to secondary reactions, such as condensation, aromatization, and the formation of recalcitrant or inhibitory compounds, which reduce biomethane production [11,12]. Therefore, the performance of a THP-AD system cannot be evaluated solely based on sCOD increase, but rather must be interpreted in terms of how hydrothermal severity redistributes carbon between bioavailable liquid-phase substrates and thermally stable solid-phase residues.

Bioelectrochemical anaerobic digestion (BEAD) is emerging as a promising approach for managing the high concentrations of soluble intermediates produced by THP [13,14,15,16,17]. By facilitating direct interspecies electron transfer (DIET) between electroactive bacteria, electrodes, and methanogens, BEAD accelerates the conversion of volatile fatty acids (VFAs) and improves process stability under elevated OLRs and when treating organic wastes containing compounds capable of inhibiting methanogenic activity [18,19,20]. In this context, THP and BEAD form a complementary pair of technologies: THP converts sludge into readily accessible substrates, while BEAD enhances the reactor’s kinetic capacity to convert these substrates into methane under short HRT operation.

Despite increasing interest in combining THP with anaerobic digestion [21,22], most studies focus on methane yield or COD removal under fixed operating conditions, without explicitly addressing the organic load–biogas production response relationship of the AD system. In practice, high-rate anaerobic digestion is governed not only by OLR, but also by the interplay between substrate accessibility and reaction kinetics. When hydrolysis remains limiting, increasing OLR leads to declining conversion efficiency and process instability. In contrast, when substrate accessibility is sufficiently enhanced, the system can transition into a kinetically controlled regime, enabling stable operation at high OLRs and short HRTs. How THP severity governs this regime transition, particularly in a BEAD reactor, is poorly understood.

In addition, in full-scale wastewater treatment plants, solid residues are separated from the liquid stream and subsequently require further processing, typically through thickening and dewatering, followed by final disposal by incineration, land application, or landfilling. Therefore, although methane recovery from the liquid phase is the primary pathway for energy production, the physicochemical properties of the residual solids, such as their higher heating value (HHV) and thermal stability, are also important determinants of overall process efficiency. Hydrothermal treatment may thus play a dual role by simultaneously enhancing liquid-phase bioenergy recovery and improving the downstream thermal management of solids.

In this study, the synergistic performance of an integrated THP-BEAD system is systematically evaluated. We investigate how hydrothermal severity (120 and 150 °C) controls organic matter solubilization, carbon partitioning, and energy demand, and how the resulting feedstocks affect BEAD performance under varying HRTs and OLRs. By jointly analyzing methane production, load-response behavior, solid-phase properties, and energy implications, this work aims to establish a regime-based operational framework for high-rate sludge digestion. This integrated approach is expected to enable compact reactor configurations, enhance energy recovery, and reduce residual solids management costs in high-density urban wastewater treatment systems.

## 2. Materials and Methods

### 2.1. Sludge Source and Preparation

Waste-activated sludge was collected from the gravity thickener of the Nanzih Wastewater Treatment Plant (Kaohsiung, Taiwan). This unit represents the final holding stage prior to anaerobic digestion; therefore, the collected sludge was considered representative of the actual feedstock used in full-scale anaerobic digesters.

After sampling, each batch of sludge was thoroughly mixed to ensure homogeneity and to eliminate phase stratification caused by particle settling during storage. The homogenized sludge was then fed into the hydrothermal pretreatment system. A 500 mL sub-sample was withdrawn from each batch for physicochemical characterization. All samples were stored at 4 °C prior to analysis and experimental use to minimize biological activity and compositional changes.

In addition to the raw sludge with a total solids content of approximately 3%, a concentrated sludge feedstock was prepared to simulate gravity-thickened sludge often used as feedstock for municipal anaerobic digesters. This was achieved by partial removal of the raw sludge liquid phase through filtration, followed by recombination of the retained solids with a controlled volume of filtrate to obtain a total solids content of approximately 4.5%. This procedure increased the total organic matter concentration while maintaining the original sludge compounds.

### 2.2. Thermal Hydrolysis Pretreatment

Hydrothermal pretreatment was conducted in a batch-operated high-pressure THP reactor with a total volume of 12 L and a maximum operating temperature and pressure of 400 °C and 250 kg cm^−2^, respectively. For each experimental run, 8 L of sludge was loaded into the reactor.

The reactor was pressurized and heated until the target temperature and a pressure of 20 kg cm^−2^ were reached. Once both conditions were achieved, the reaction was maintained for 15 min to ensure sufficient hydrothermal conversion. After completion of the pretreatment, the reactor was allowed to cool to room temperature.

The treated sludge was subjected to solid-liquid separation using a mesh filter with a pore size of 0.02 mm to remove large particulates. The resulting liquid fraction, containing fine suspended solids and soluble organics, was directly used as the feedstock (influent) for BEAD reactor operation. Sub-samples were withdrawn for physicochemical analysis, and the remaining THP liquor was stored at 4 °C prior to BEAD tests.

The operating temperatures of the thermal hydrolysis pretreatment tests and the corresponding sCOD concentrations of the treated liquors (process water), which were used to select the THP conditions for BEAD reactor operation, are summarized in Table 1.

### 2.3. BEAD Reactor Design and Operation

The bioelectrochemical anaerobic digestion (BEAD) reactor was designed based on the concept of an upflow anaerobic sludge blanket (UASB) reactor [23]. Accordingly, the BEAD reactor consisted of a vertical PVC column with an internal diameter of 200 mm (cross-sectional area of 314 cm^2^) and five sampling ports along the reactor height.

Two pairs of electrodes (two anodes and two cathodes) were installed in the reactor. The electrodes were constructed from conductive biorings made of polypropylene blended with 15 wt% Carbon Black (CB), forming a flow-through three-dimensional conductive matrix for biofilm attachment and charge transfer. Electrical separation between anodes and cathodes was achieved using non-conductive PVC mesh spacers (approximate thickness of 1 mm). Titanium wires connected to carbon felt strips placed in the middle of each electrode compartment were used as current collectors to ensure uniform current distribution across the electrode beds. The electrodes were connected to a power supply (KORAD KD3005P, Dongguan KORAD Technology Co., Ltd., Dongguan, China) and operated with periodic polarity reversal.

The total volume of the electrode zone was approximately 12 L and was defined as the effective reactor volume (L_R_) for all performance calculations, including OLR and volumetric methane production. The total liquid volume in the reactor was approximately 17 L, with a headspace of about 3 L.

**Figure 1:**
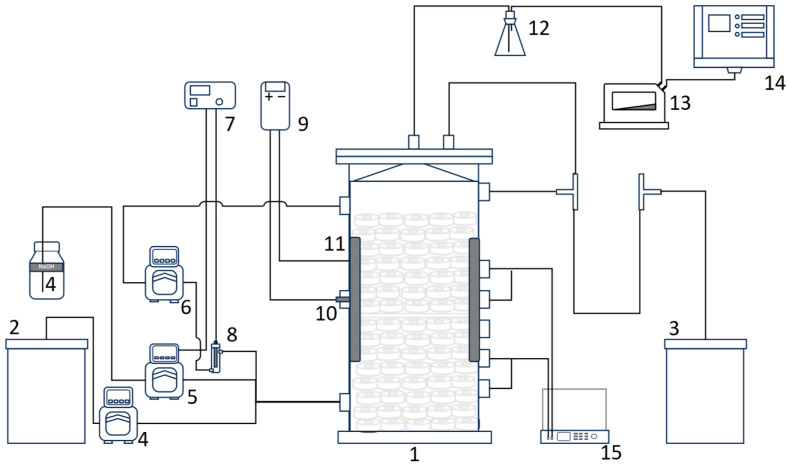
Schematic of the laboratory-scale BEAD reactor integrated with process monitoring and control systems. The setup consisted of a (1) BEAD reactor, (2) feed tank, (3) overflow tank, (4) feed pump, (5) NaOH dosing pump, (6) recirculation pump, (7) pH controller, (8) pH meter, (9) temperature controller, (10) temperature sensor, (11) heater, (12) gas collection flask, (13) gas flow meter, (14) methane concentration analyzer, and (15) power supply.

An external recirculation loop was operated at 5.7 L h^−1^, corresponding to an upflow velocity of approximately 17 cm h^−1^, to provide hydraulic mixing and enhance mass transfer within the electrode zone. A schematic of the reactor is shown in Figure 1.

The BEAD reactor was continuously fed with the liquid fraction of sludge after hydrothermal pretreatment. The reactor was equipped with temperature and pH control systems. Temperature was maintained at 30 °C, unless specified otherwise. An automatic pH control system consisting of a pH probe installed in the recirculation loop, a controller, and a peristaltic pump was used. A 0.5 M NaOH solution was used to maintain the reactor pH within the range of 6.9–7.2.

During start-up, the reactor was operated at a long HRT (24 days) to develop a stable anaerobic electroactive microbial community. Once stable performance was observed, as indicated by stable pH, biogas production, and methane concentration, the HRT was gradually reduced to evaluate reactor performance under progressively increasing organic loads.

The target HRT values were sequentially set to 24, 12, 6, 3, 2.5, 2, and 1.5 days. Due to minor flow variations and pH control, the actual HRT values deviated slightly from these setpoints. The operating phases were conducted sequentially in time by stepwise reduction in HRT. The order of HRT values in Table 2 reflects the actual experimental timeline. To reduce HRT at each phase, the influent flow rate was increased accordingly. Because the COD concentration of the THP liquor varied with pretreatment temperature and concentration, the resulting OLR was governed by both HRT and influent COD. The detailed operating conditions are summarized in Table 2, in which soluble COD values used for OLR calculation are denoted as sOLR.

Throughout the operation, influent flow rate, pH, temperature, biogas production rate, methane concentration, and electrochemical parameters were continuously monitored and recorded at 4 h intervals. Each operating condition was maintained for 7 days before the data were used for performance evaluation.

### 2.4. Analytical Methods and Calculations

The COD determination of the liquid samples was carried out according to USEPA410.4 [24]. For total COD (tCOD) concentration measurements, samples were analyzed after appropriate dilution. For sCOD, samples were filtered through 0.22 µm syringe filters prior to analysis. Approximately 2 mL of sample was added to high-range COD digestion vials (HACH) and digested at 150 °C for 2 h using a COD reactor (CR25, Rocker Scientific Co., Ltd., New Taipei City, Taiwan). After digestion, COD was measured using a spectrophotometric COD analyzer (CD200, Rocker Scientific Co., Ltd., New Taipei City, Taiwan). All analyses were performed in duplicate and reported as average values.

The higher heating value (HHV) of the raw sludge and hydrothermally treated solids was determined using a bomb calorimeter (C3000, IKA Werke GmbH & Co. KG, Staufen, Germany) [25].

Specific volumetric methane yield (*SMY*) was expressed in L of produced CH_4_ per L of hydrothermally pretreated liquor fed to the reactor:

(1) $$SMY=\frac{F_{CH4}}{F_{THP}},$$

where *F_CH_*_4_ is the methane production rate (L d^−1^) and *F_THP_* is the hydrothermally pretreated feedstock flow rate (L d^−1^).

COD-based methane yield (*Y_CH_*_4_) normalized to COD consumption was calculated as

(2) $$Y_{CH4}=\frac{F_{CH4}}{F_{THP}\left(sCOD_{in}-sCOD_{out}\right)},$$

where *sCOD_in_* and *sCOD_out_* are the influent and effluent sCOD concentrations (g L^−1^).

## 3. Results and Discussion

### 3.1. Control of Reactor Loading by Progressive HRT Ramping

Hydrothermal pretreatment temperature exerted a clear influence on the sCOD concentration of the feedstock. As summarized in Table 1, sCOD increased progressively with increasing pretreatment temperature over the investigated range (120–210 °C), showing an approximately linear increase of sCOD within the tested conditions. This temperature-dependent increase in sCOD defined the COD concentration of the influent stream entering the BEAD reactor.

While the degree of organic solids solubilization increased with increasing THP temperature, the amount of soluble compounds inhibiting methanogenic populations, mainly Maillard reaction products such as pyrazines and pyridines, was also expected to increase [26]. Additionally, decomposition of lignin and humic compounds leads to phenol formation [27]. Energy consumption for sludge heating to the target THP temperature also increases proportionally with the THP temperature setpoint. Several previous studies have used hydrothermal pretreatment temperatures ranging from 150 to 210 °C, with one study suggesting an optimal temperature of 199 °C [28]. However, these studies did not consider energy requirements for THP reactor operation. Consequently, in the present study, two distinctly different THP temperatures of 120 °C and 150 °C were selected to represent a mild THP treatment, which minimizes the production of inhibitory soluble products and reduces energy consumption, and a more severe treatment, respectively.

For each selected THP temperature, the BEAD reactor was operated at several OLRs obtained using a stepwise reduction in HRT designed to study reactor performance and stability under progressively increasing organic loads. Operating conditions in the following discussion correspond to the chronological sequence of the experiment.

**Figure 2:**
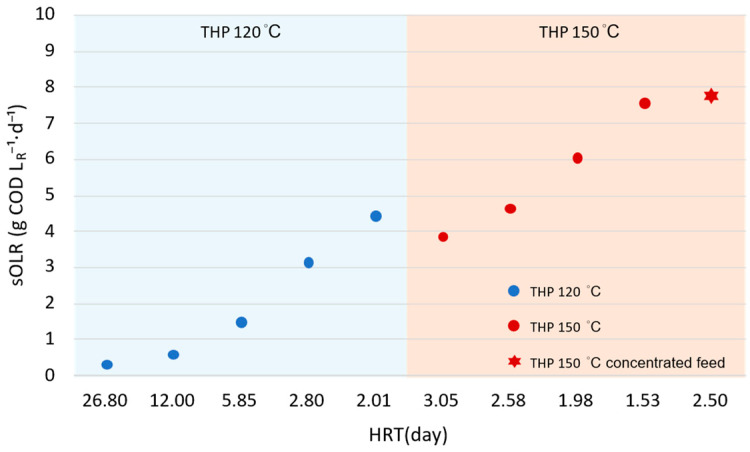
Relationship between hydraulic retention time (HRT) and soluble organic loading rate (sOLR) during operation of the THP-BEAD system. Data points follow the chronological sequence of HRT ramping. Blue circles represent 120 °C pretreatment, red circles represent 150 °C pretreatment, and the red star indicates operation with concentrated feed (Total Solids ≈ 4.5%).

Because the influent COD concentration was governed by hydrothermal pretreatment temperature and sludge concentration, the resulting sOLR was controlled by both influent sCOD concentration and HRT. Figure 2 illustrates the relationship between HRT and sOLR for the feedstocks obtained at 120 °C and 150 °C pretreatment temperatures, which were subsequently used for BEAD reactor operation.

For the 120 °C pretreatment, decreasing the HRT from 26.8 to 2.01 days led to an increase in sOLR from 0.30 to 4.42 g $L_{R}^{-1}$·d^−1^. The 150 °C pretreatment produced higher influent sCOD concentrations, resulting in a higher sOLR range under comparable HRT conditions. Specifically, sOLR increased from 3.85 g $L_{R}^{-1}$·d^−1^ at an HRT of 3.05 days to 7.54 g $L_{R}^{-1}$·d^−1^ at an HRT of 1.53 days. When concentrated sludge was subjected to 150 °C pretreatment, an even higher sOLR of 7.75 g $L_{R}^{-1}$·d^−1^ was achieved at an HRT of 2.5 days.

These results demonstrate that HRT functioned as the primary operational control parameter for regulating reactor loading, while hydrothermal pretreatment severity (temperature) governed the influent COD concentration [29,30]. Consequently, the two pretreatment temperatures established distinct loading regimes: the 120 °C series corresponded to a low-to-moderate sOLR range (0.30–4.42 g $L_{R}^{-1}$·d^−1^), whereas the 150 °C series enabled operation under substantially higher sOLR conditions (3.85–7.75 g $L_{R}^{-1}$·d^−1^) at shorter HRTs.

The longest HRT (26.8 days) in Figure 2 corresponds to the reactor start-up and microbial adaptation phase and is therefore excluded from the steady-state performance analysis in the subsequent discussion.

### 3.2. Performance of the 120 °C Hydrothermally Pretreated Sludge Under Increasing sOLR

**Figure 3:**
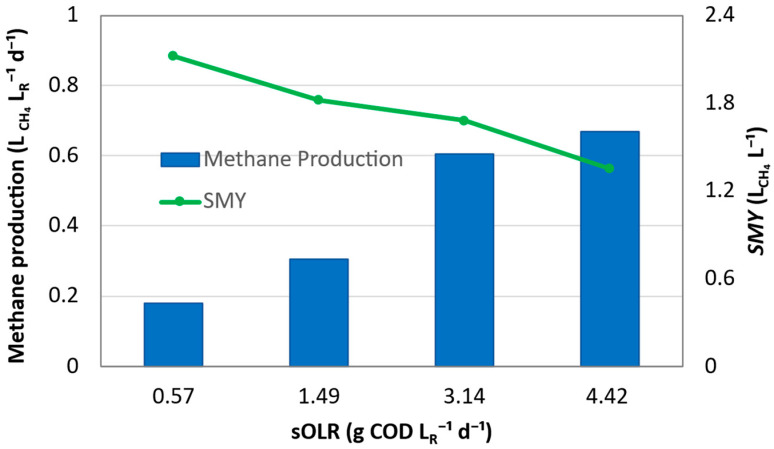
Effect of sOLR on volumetric methane production and specific methane yield (*SMY*) of the bioelectrochemically assisted anaerobic digestion (BEAD) reactor fed with sludge hydrothermally pretreated at 120 °C. The sOLR was increased by progressively reducing the HRT from 12.0 to 2.01 days. Bars represent volumetric methane production (L_CH4_ $L_{R}^{-1}$·d^−1^), and the line represents *SMY* (L_CH4_ L^−1^). The opposing trends indicate increasing methane throughput but decreasing substrate conversion efficiency with increasing sOLR.

The methane production performance of the BEAD reactor fed with sludge pretreated at 120 °C is shown in Figure 3 as a function of sOLR. As the HRT was progressively reduced from 12.0 to 2.0 days, sOLR increased from 0.57 to 4.42 g $L_{R}^{-1}$·d^−1^, and the volumetric methane production rate increased from 0.18 to 0.67 L_CH4_ $L_{R}^{-1}$·d^−1^. This indicates that the reactor was able to accommodate higher substrate loading by converting more organic materials into methane, consistent with enhanced substrate supply resulting from hydrothermal pretreatment [31].

However, despite the increase in volumetric methane production, the specific volumetric methane yield (*SMY*, Equation (1)) decreased monotonously from 2.10 to 1.35 L L^−1^ as sOLR increased. This divergence between volumetric methane production and *SMY* reflects a progressive loss of conversion efficiency at higher loading conditions and suggests that a significant fraction of the THP process water requires further hydrolysis to facilitate methane production. At low sOLR and therefore sufficiently long retention times, a larger fraction of the soluble organics released by pretreatment could be converted into methane, whereas at higher sOLR, the insufficient retention time resulted in incomplete substrate utilization. At the same time, for the COD-based methane yield (*Y_CH4_*, Equation (2)), the values ranged from 0.27 to 0.36 L_CH4_ g^−1^ sCOD for all tested sOLR conditions without a clear monotonic trend. This suggests that, although specific volumetric methane yield declined with increasing sOLR, the intrinsic methane conversion efficiency of the consumed COD remained largely comparable over the investigated loading range. In contrast, the overall soluble COD removal decreased progressively from 98.2% to 79.7% as HRT was shortened and sOLR increased, suggesting that an increasing fraction of the feedstock could not be accessed by the anaerobic methanogenic consortium and converted within the applied retention time.

This behavior is characteristic of a hydrolysis-limited regime, in which increasing the loading rate leads to higher volumetric throughput but lower substrate utilization efficiency [32,33]. Although hydrothermal pretreatment at 120 °C increased sludge solubilization, the accessibility and biodegradability of the released organics remained insufficient to sustain efficient methanogenesis under short HRTs. This limitation is also reflected in the relatively shallow slope of the methane production–sOLR relationship (discussed below), indicating a decreasing methane recovery per unit increase in sOLR.

### 3.3. Performance of the 150 °C Hydrothermally Pretreated Sludge Under High sOLR

**Figure 4:**
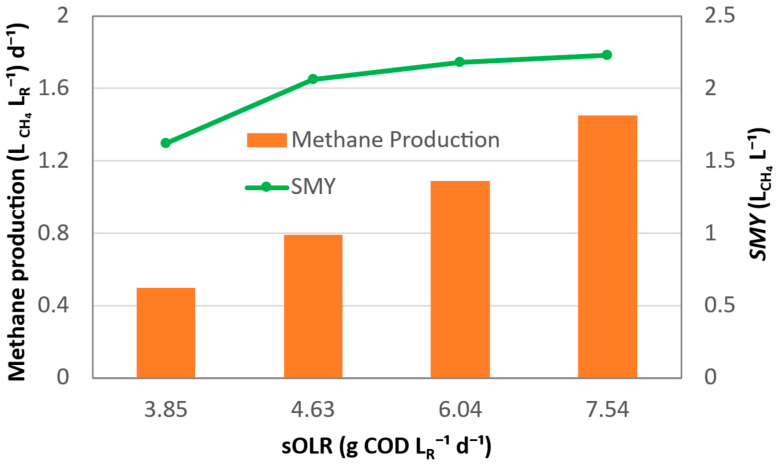
Effect of sOLR on volumetric methane production and *SMY* of the BEAD reactor fed with sludge hydrothermally pretreated at 150 °C. The sOLR was increased by progressively reducing the HRT from 3.05 to 1.53 days. Bars represent volumetric methane production (L_CH4_ $L_{R}^{-1}$·d^−1^), and the line represents *SMY* (L_CH4_ L^−1^).

The performance of the BEAD reactor fed with sludge hydrothermally pretreated at 150 °C is presented in Figure 4 as a function of the sOLR. By progressively decreasing the HRT from 3.05 to 1.53 days, the sOLR increased from 3.85 to 7.54 g $L_{R}^{-1}$·d^−1^, resulting in a consistent near-linear increase in volumetric methane production from 0.53 to 1.46 L_CH4_ $L_{R}^{-1}$·d^−1^. Compared with the 120 °C pretreatment experiment, a steeper slope of the trend line was obtained, indicating that the higher hydrothermal severity markedly enhanced the ability of the reactor to sustain high methane production under short HRT operation.

In contrast to the 120 °C pretreatment, no decline in *SMY* was observed with increasing sOLR at 150 °C. Instead, *SMY* increased from 1.62 to 2.23 L_CH4_ L^−1^ as sOLR rose from 3.85 to 7.54 g COD L^−1^·d^−1^ (Figure 4). The concurrent increase in both volumetric methane production and *SMY* suggests that methanogenic activity was not limited by feedstock hydrolysis or mass transfer, even at the highest sOLR tested. Rather, hydrothermal pretreatment at 150 °C resulted in highly biodegradable soluble organic substrates that did not require a hydrolysis step and enabled methane production to closely follow the increasing supply of substrate [34].

*Y_CH_*_4_ values ranged from approximately 0.25 to 0.36 ± 0.1 L g^−1^ for all tested sOLR values, and no decrease in the COD-based methane yield with increasing sOLR was observed. Interestingly, *Y_CH_*_4_ values observed during reactor operation using feedstock pretreated at both 120 °C and 150 °C were close to the theoretical limit, while most AD tests using THP liquid have reported significantly lower yields and longer treatment times. For example, methane production of 415.4 mL g^−1^ of volatile solids (corresponding to approximately 0.3 L g^−1^ COD) and a treatment time of 30 days were reported by Senol et al. [35] based on biochemical methane potential (BMP) tests using pistachio skin feedstock. Similar methane yields were reported in AD studies using hydrothermally pretreated waste-activated sludge [36] and food waste [37,38] feedstocks. At the same time, high methane yields have also been reported in several studies involving bioelectrochemically enhanced anaerobic reactors [14], which suggests that a combination of bioelectrochemical and conventional pathways leads to enhanced methane production.

Meanwhile, soluble COD removal remained in the range of approximately 75–81%, indicating that although hydrolysis and substrate accessibility were improved at 150 °C, the overall extent of COD removal was still influenced by short HRTs and the kinetics of methane production by methanogenic microorganisms rather than by hydrolysis [39]. 

At least in part, such robust performance can be attributed to the development of an electroactive biofilm on the conductive biorings forming the anode and cathode compartments of the BEAD reactor. This biofilm likely facilitated electron transfer and enhanced syntrophic interactions between microbial populations. Several previous studies have demonstrated the benefits of incorporating microbial electrolysis cell (MEC) bioelectrodes into anaerobic reactors and operating the resulting bioelectrochemical anaerobic digestion systems at applied voltages below the onset of water electrolysis [13,14,16,40,41,42]. These studies have highlighted BEAD advantages such as increased methane yield and improved reactor stability when operating on feedstocks that may inhibit methanogenic populations, particularly acetoclastic methanogens.

### 3.4. Impact of Hydrothermal Treatment Severity on BEAD Reactor Operational Regime

**Figure 5:**
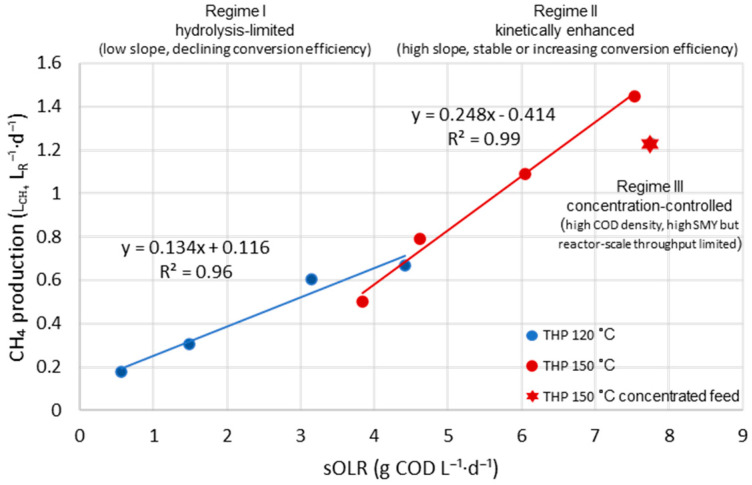
Regime map of the THP–BEAD process showing the dependence of volumetric methane production on sOLR at different hydrothermal pretreatment severities and feed concentrations. Solid lines represent linear regressions for Regime 1 (blue) and Regime II (red).

The relationship between volumetric methane production and sOLR was examined to elucidate how THP severity governs the kinetic response of the BEAD reactor under progressively intensified operating conditions. As shown in Figure 5, methane production exhibited an approximately linear dependence on sOLR for both pretreatment temperatures within the investigated range of sOLR values. However, the distinctly different slopes of the trendlines reveal a fundamental shift in the factors affecting methane production as a function of THP severity.

For sludge pretreated at 120 °C, methane production followed the regression equation y = 0.134x + 0.117 (R^2^ = 0.96), indicating a relatively low sensitivity (small slope value) of volumetric methane production to increasing sOLR. Such a shallow slope is consistent with the declining specific methane yield observed at shortened HRTs, demonstrating that although higher organic loading increased volumetric methane production, the released soluble substrates were not sufficiently accessible to sustain efficient conversion under high-rate operation. Consequently, the 120 °C pretreatment corresponds to a hydrolysis-limited regime, in which substrate accessibility and microbial hydrolysis kinetics limit the effective utilization of the feedstock [43].

In contrast, sludge pretreated at 150 °C exhibited a substantially steeper slope in the linear relationship between methane production and sOLR, described by y = 0.248x − 0.414 (R^2^ = 0.99). Compared with the results obtained at a pretreatment temperature of 120 °C, the approximately 85% increase in slope indicates that solubilized organics were converted into methane much more effectively. This behavior, together with the stable or increasing specific volumetric methane yield observed under decreasing HRTs, identifies a substrate-limited operating regime, in which THP-generated substrates could be rapidly and efficiently metabolized by the bioelectrochemical anaerobic digestion system.

At the highest COD concentration achieved using concentrated feed at 150 °C (Phase 10), the volumetric methane production rate was lower than that observed under the shortest HRT in Phase 9. However, this difference should be interpreted primarily in the context of hydraulic retention time rather than substrate concentration alone. When compared at similar HRTs, the concentrated-feed operation in Phase 10 (HRT = 2.5 days) exhibited substantially higher methane production and specific methane yield than the operation using the non-concentrated feedstock at a comparable HRT (Phase 7, HRT = 2.58 days). This comparison indicates that increased COD load and concentration in Phase 10 did not impair reactor performance, but instead enhanced the effective utilization of soluble organics. The apparent deviation from the linear methane production vs. sOLR trend therefore reflects the combined influence of COD concentration and HRT, rather than the concentration-induced inhibition, which would correspond to the organic overload regime. Table 3 provides a summary of operating conditions and corresponding performance parameters for both pretreatment temperatures.

Taken together, these results demonstrate that increasing THP severity from 120 to 150 °C does not merely increase soluble COD concentration, but fundamentally alters the load-response behavior of the BEAD reactor. With the change in pretreatment temperature, the system transitioned from a hydrolysis-limited regime to a kinetically enhanced regime capable of sustaining high methane productivity under short-HRT operation [44]. Within this framework, HRT remains the primary operational control parameter, while feedstock concentration modulates COD density without inherently compromising reactor performance. This regime-based interpretation provides a mechanistic basis for defining practical operating windows for high-rate THP-BEAD systems.

### 3.5. Electrochemical Current Response Under Periodic Polarity Reversal

During BEAD operation, the cell voltage was maintained at a constant value of 1.2 V, while electrode polarity was periodically reversed every 2 min (with 15 s between power supply re-connections) using an external switching device. This mode of operation led to a reproducible transient current response within each “on” cycle, characterized by an instantaneous current peak immediately following polarity reversal and power supply re-connection, followed by a gradual decay toward a quasi-steady-state current level. Accordingly, the maximum (initial) current (*I_max_*) and minimum (at the end of the approaching steady state) current (*I_min_*) values were recorded. These values can be associated with the electroactive biofilm density and the bioelectrochemical activity of the anodophilic and cathodophilic electroactive microbial populations. Indeed, in a BEAD reactor, the formation of a biofilm on the electrode surface increases double-layer capacitance and enhances the steady-state current of the electrodes. When the electrode polarity is periodically reversed, or the power supply is intermittently disconnected and then re-connected, a transient high current is observed due to the double-layer capacitance of the biofilm. This current gradually stabilizes at a lower level, reflecting the metabolic activity of electroactive microorganisms.

By monitoring both the *I_max_* and *I_min_* currents, biofilm thickness (or metabolic state) and electroactive activity can be assessed, respectively. Notably, under applied voltage conditions, anodophilic bacteria facilitate substrate oxidation at the anode, while cathodophilic electroactive bacteria produce hydrogen at the cathode. *I_min_* values are expected to reflect these microbial activities. Notably, hydrogen is converted into methane by hydrogenotrophic methanogens, which also utilize dissolved carbon dioxide. A thicker biofilm is expected to lead to higher overall microbial activity, reflected in larger *I_max_* values. Moreover, reactor overload or microbial activity inhibition is expected to result in a decline in both *I_max_* and *I_min_* values.

**Figure 6:**
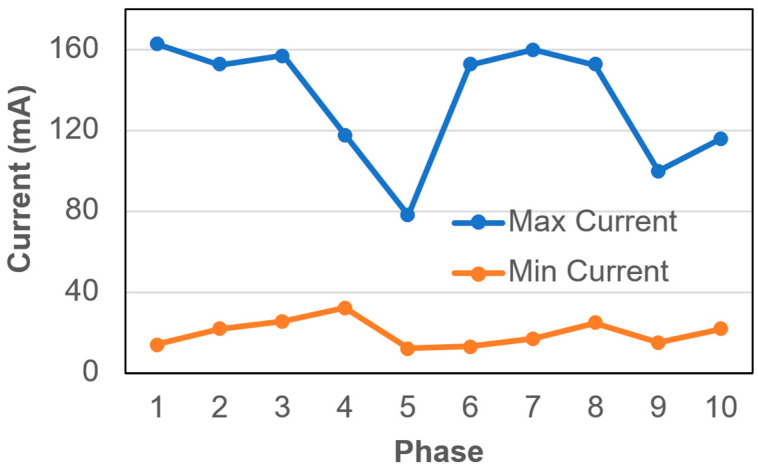
Maximum and minimum current recorded during BEAD operation under a constant applied voltage of 1.2 V with periodic polarity reversal every 2 min. Transient current peaks occurred immediately after polarity switching, followed by decay to quasi-steady current levels, reflecting the electrochemical response of the reactor under different operating phases.

As shown in Figure 6, both *I_max_* and *I_min_* varied systematically across the operating phases in response to changes in HRT, pretreatment temperature, and feedstock concentration. Under the 120 °C pretreatment (Phases 1–5), *I_max_* remained relatively stable at approximately 150–165 mA during reactor operation at long to moderate HRTs (Phases 1–3). However, a pronounced decrease in both *I_max_* and *I_min_* was observed when the HRT was further reduced (Phase 5), indicating a reduction in bioelectrochemical activity under increased sOLR.

Following the transition to 150 °C pretreatment of the feedstock (Phases 6–8), the reactor exhibited a rapid recovery of electrochemical activity, likely due to longer HRT values in Phases 6 and 7. Both *I_max_* and *I_min_* values increased to levels comparable to or higher than those observed during Phases 1–3, despite operation at substantially shorter HRTs. This recovery suggests that the electroactive biofilm adapted effectively to the increased availability of readily biodegradable substrates produced by higher-severity thermal hydrolysis at 150 °C.

At the shortest HRT using non-concentrated feedstock (Phase 9), a moderate decrease in current, similar to that in Phase 5, was observed once again, although the magnitude of decline was less pronounced. At the highest sOLR (concentrated feedstock pretreated at 150 °C) in Phase 10, *I_max_* and *I_min_* values increased again, indicating that increased COD concentration and, therefore, availability of biodegradable substrates increase bioelectrochemical activity if sufficient retention time is provided.

Overall, the observed current profiles demonstrate that the BEAD reactor current is sensitive to changes in operating conditions, with current values declining under experimental conditions corresponding to short retention times and declining COD removal efficiency. Each time the bioelectrochemical activity promptly recovered once the HRT was increased, i.e., the current decrease was indicative of approaching organic overload and decreasing COD removal efficiency, even before it was detected based on methane production. While current changes reflect combined contributions from biofilm capacitance, electrochemical reactions, and mass-transfer limitations within the biofilm, consistent current recovery when using feedstock pretreated at 150 °C supports the choice of this pretreatment temperature.

### 3.6. THP–BEAD Comparison with Conventional Sludge Digestion

To benchmark the performance of the THP-BEAD system, reactor operation during the substrate-limited regime corresponding to feedstock pretreatment at 150 °C was compared with the conventional anaerobic digester operated at the Nanzih municipal wastewater treatment plant (WWTP). Notably, both the feedstock sludge and the inoculum used for THP and BEAD setups operation were obtained from the same municipal wastewater treatment plant, allowing the comparison to be based on the site-specific anaerobic digestion baseline. Conventional sludge digesters are generally designed for stable operation under long hydraulic retention times (HRTs), commonly on the order of 3–4 weeks, and relatively low volumetric loading rates. Under such conditions, methane production is still limited by sludge hydrolysis, and reactor footprint requirements are substantial.

In the THP-BEAD study, thermal hydrolysis pretreatment at 150 °C enabled stable methane production at substantially reduced HRTs, while sustaining high sOLR values, i.e., stable anaerobic digestion can be maintained at significantly higher OLRs when thermal hydrolysis is combined with BEAD. The observed methane production rates therefore indicate that the combined THP-BEAD approach enables stable, high-rate sludge digestion under significantly shorter HRTs.

From an engineering perspective, the ability to sustain a high rate of biogas production at a short HRT suggests potential advantages in terms of reactor volume reduction, even when treating concentrated sludge. Such a configuration may be particularly relevant for space-constrained wastewater treatment facilities or for a retrofitting scenario, where incremental capacity expansion is required. Importantly, the present results do not imply direct replacement of conventional anaerobic digesters, but instead demonstrate the feasibility of complementary high-rate digestion modules integrated within existing treatment infrastructures.

Overall, this comparison underscores that the primary contribution of the THP-BEAD system lies in changing the relationship between loading rate and reactor kinetics. By shifting the digestion process from a hydrolysis-limited regime to a substrate-limited high-rate regime, the system provides an engineering basis for evaluating how far conventional sludge digestion processes may be intensified following feedstock pretreatment combined with enhanced anaerobic digestion methods.

### 3.7. Fate of the Solid Fraction and Downstream Process Implications

To elucidate the effects of THP severity on the physicochemical transformation of sewage sludge, solid-phase characterization was conducted to complement the liquid-phase analyses. It should be emphasized that the temperature conditions selected for solid-phase analysis were not intended to fully mirror those used in the liquid-phase experiments, as the two analyses addressed distinct research objectives within the integrated THP–BEAD process.

Following pretreatment, sludge is separated into a liquid fraction (process water) for further treatment in the BEAD reactor and a solid fraction (hydrochar) that must be managed by a downstream process. In large-scale wastewater treatment systems, municipal sludge is often subjected to incineration as a final disposal method [45,46]. Therefore, although the primary objective of this study is methane recovery from the liquid phase, the characteristics of the residual solid fraction remain critically important for evaluating the overall plant operation, as they directly influence solids handling, disposal strategies, energy recovery options, and treatment costs.

Throughout BEAD reactor operation, THP at 120 and 150 °C was systematically investigated to evaluate organic solubilization, substrate biodegradability, and anaerobic digestion performance, representing conditions relevant to biological processing. In contrast, the solid-phase analysis focused on THP residues obtained at a broader range of temperatures, including higher thermal severities, to assess changes in energy-related characteristics. Accordingly, samples produced at 150, 180, and 210 °C were selected for solid-phase evaluation, while 120 °C was not included in the present solid-phase analysis.

As summarized in Table 4, the higher heating value (HHV) of the solid fraction increased from 12,034 kJ kg^−1^ for raw sludge to 12,888 kJ kg^−1^ after THP at 150 °C. A pronounced increase in HHV was observed at 180 °C, reaching 15,725 kJ kg^−1^. When the pretreatment temperature was further increased to 210 °C, the HHV slightly decreased to 15,191 kJ kg^−1^, while remaining higher than that of both raw sludge and the solids obtained at 150 °C.

These results indicate that the THP temperature influences the energy content of the solid fraction. Given that incineration is a common final disposal pathway for sewage sludge, a higher HHV of the solid fraction may be beneficial by providing greater energy availability during combustion-based treatment, potentially contributing to more stable incineration operation or reducing the reliance on auxiliary fuels [47]. Considering the integrated THP-BEAD system, a pretreatment temperature range of 150–180 °C can be considered a practical operating window in which enhanced solid fuel properties are achieved while limiting, but not necessarily eliminating, potential inhibition of methanogenesis associated with higher thermal severity.

### 3.8. Energy Performance of the THP-BEAD System

The THP-BEAD system adopts a fundamentally different energy–process trade-off from conventional anaerobic digestion. Rather than maximizing methane yield per unit of feed, the system is designed to maximize reactor throughput, volumetric energy productivity, and organic matter conversion efficiency by using hydrothermal pretreatment to shift the digestion regime from hydrolysis-limited to substrate-limited operation. In addition to increasing methane production rates, this regime shift also leads to a higher fraction of volatile solids being biologically converted, thereby reducing the amount of residual solids that must be handled, dewatered, and disposed of downstream.

In Phase 7 (150 °C pretreatment, HRT = 2.58 days), the BEAD reactor achieved a volumetric methane production rate of 0.79 L_CH4_ $L_{R}^{-1}$·d^−1^, which is more than one order of magnitude higher than that of a full-scale municipal anaerobic digester at the Nanzih WWTP (0.049 L_CH4_ $L_{R}^{-1}$·d^−1^, Table 5). At the same time, the hydraulic retention time was reduced from 55.5 days to 2.58 days, implying that the same sludge treatment capacity could, in principle, be achieved with a digester volume more than 20-fold smaller. Although a pilot-scale demonstration is required to confirm such performance, it can already be emphasized that any reduction in residence time directly reduces the energy required for maintaining reactor temperature, mixing, and pumping, because the feedstock remains in the reactor for a much shorter period.

The THP-BEAD configuration also enables an efficient thermal coupling of the two systems. Hydrothermally pretreated sludge leaves the THP unit at elevated temperature and can be fed directly to the BEAD reactor (after solids removal), or temporarily stored in an insulated buffer tank before feeding according to the reactor operating schedule. This substantially reduces, or even eliminates, the need for external heating of the anaerobic reactor. In this configuration, thermal energy invested in THP is not lost, but at least partially retained within the process to support the operating temperature of the downstream anaerobic treatment. From a system perspective, this corresponds to reallocating the energy originally required for long-term temperature maintenance of a large anaerobic reactor to a short-duration, high-temperature THP heating, enabled by continuous operation of the integrated setup and feed scheduling.

The energy demand of THP can be evaluated from two complementary perspectives. Based on the present batch-mode experimental system, the measured electricity consumption corresponds to approximately 0.65 kWh per liter of hydrothermally pretreated sludge, which represents a conservative upper boundary associated with non-integrated laboratory operation. To approximate a more realistic industrial implementation, a heat recovery model can be applied. Because sludge has a very high moisture content (above 90%), its thermal behavior was approximated using the thermophysical properties of water for energy calculations. Assuming 75% heat recovery (k = 0.75) between successive THP cycles, the effective external thermal energy required to heat the feed from ambient temperature to the THP operating temperature is reduced to 25% of the theoretical heating requirement, corresponding to 0.16 kWh L^−1^.

Importantly, the role of the THP energy input in the THP-BEAD system is not to directly generate methane, but to cause a shift from hydrolysis-limited to substrate-limited reactor operation at significantly higher organic loading rates and much shorter retention times. In conventional anaerobic digestion, energy is continuously used to maintain a very large reactor at mesophilic temperatures, with the reactor operating at an HRT of several weeks. In contrast, the THP-BEAD system concentrates energy input into a short, high-temperature pretreatment step, in exchange for a substantially smaller reactor with significantly reduced or even negligible external heating demand, and higher volumetric methane productivity due to the thermal hydrolysis pretreatment essentially resolving hydrolysis limitations and facilitating anaerobic digestion in a lower range of mesophilic temperatures [48]. Furthermore, BEAD enhances anaerobic digestion at lower temperatures [49]. From an engineering perspective, this represents a transfer of energy demand from long-duration, low-intensity reactor operation to short-duration, high-intensity thermal pretreatment. When heat recovery and thermal integration between THP and BEAD are considered, this transfer provides a practical pathway to develop a high-rate sludge stabilization and methane production process without a proportional increase in overall energy consumption, establishing THP-BEAD as a thermally driven, high-intensity waste-to-energy platform.

## 4. Conclusions

This study evaluated the performance of an integrated THP–BEAD system for sewage sludge treatment under progressively increasing organic loads and at two distinctly different pretreatment temperatures of 120 °C and 150 °C. By coupling stepwise reduction in HRT with controlled variation in pretreatment severity, the OLR–methane production response of the BEAD reactor was analyzed in terms of distinct operating regimes.

At a pretreatment temperature of 120 °C, the BEAD reactor performance was governed by a hydrolysis-limited regime, in which increasing sOLR increased volumetric methane production but reduced conversion efficiency at short HRTs. In contrast, increasing THP severity to 150 °C shifted the system into a substrate-limited regime, characterized by a substantially stronger dependence of methane production on sOLR and stable or improved specific methane yield despite HRT reduction. This transition indicates that appropriate THP severity effectively aligns substrate accessibility with the enhanced kinetic capacity provided by BEAD.

Analysis of the THP solid fraction (hydrochar) produced showed that higher THP temperatures increased the higher heating value of residual solids, providing insight into downstream sludge management, while liquid phase methane recovery remained the primary energy production pathway.

While integration of THP with anaerobic digestion has been previously suggested, it was observed that although increasing THP temperature improves solids solubilization and produces higher quality hydrochar, higher temperatures also increase the production of compounds (e.g., Maillard reaction products, phenols) that inhibit methanogens. As a result, methane production declines. Additionally, the relatively high temperature of anaerobic digestion increases energy consumption for reactor heating, which is particularly significant in temperate climates. Bioelectrochemical anaerobic digestion has been shown to improve reactor stability in the presence of inhibitory compounds compared with conventional AD. Furthermore, it can operate in a lower range of mesophilic temperatures. Thus, the combined THP-BEAD process is expected to enable a higher degree of sludge solubilization and improved hydrochar quality, while reducing the energy required for biogas production.

From an engineering perspective, the operating conditions achieved in this study extend beyond those typically applied in conventional municipal anaerobic digesters, which commonly operate at long HRTs and low volumetric loading rates. Rather than aiming to replace existing infrastructure, the THP-BEAD configuration demonstrates the potential for complementary high-rate operation to reduce reactor footprint and/or handle concentrated sludge streams. Overall, the results show that THP severity governs the kinetic regime of digestion, with HRT as the primary operational control parameter and pretreatment severity and feed concentration defining achievable loading windows. This regime-based framework provides practical guidance for the design and operation of compact, high-throughput sludge digestion systems in space-constrained wastewater treatment facilities.

## Figures and Tables

**Table 1:** Impact of thermal hydrolysis pretreatment (THP) temperature on soluble chemical oxygen demand (sCOD) concentration of sludge-derived liquor (process water).

Temperature (°C)	sCOD (g L^−1^)
120	9.24
150	10.40
180	11.61
210	12.50
150 *	16.62

* concentrated sludge.

**Table 2:** Experimental operating conditions of the anaerobic reactor fed with THP liquor.

THP Conditions	Phase	Target HRT (Day)	Actual HRT (Day)	sOLR (g_COD_ $L_{R}^{-1}$·d^−1^)
120 °C; 20 kg cm^−2^; 15 min	1 *	24	26.8	0.30
	2	12	12.0	0.57
	3	6	5.85	1.49
	4	3	2.80	3.14
	5	2	2.01	4.42
150 °C; 20 kg cm^−2^; 15 min	6	3	3.05	3.85
	7	2.5	2.58	4.63
	8	2	1.98	6.04
	9	1.5	1.53	7.54
	10 **	2.5	2.50	7.75

* The longest-HRT, low-OLR condition was used for reactor start-up and microbial adaptation. This phase is therefore not included in the steady-state performance analysis. ** THP of concentrated sludge.

**Table 3:** Operating conditions and measured performance parameters of the BEAD reactor operated on THP liquid obtained at two different temperatures.

THP Condition (°C)	Phase	HRT (Day)	sOLR (g $L_{R}^{-1}$·d^−1^)	Methane Production (L $L_{R}^{-1}$·d^−1^)	*SMY* (L L^−1^)	*Y_CH_*_4_ (L g^−1^ sCOD)	sCOD Removal (%)
120 °C 20 kg cm^−2^ 15 min	1	26.8	0.3	0.1	2.59	0.52	91.68
	2	12	0.57	0.18	2.12	0.33	98.21
	3	5.85	1.49	0.31	1.82	0.27	92.56
	4	2.8	3.14	0.61	1.68	0.34	85.01
	5	2.01	4.42	0.67	1.35	0.36	79.70
150 °C 20 kg cm^−2^ 15 min	6	3.05	3.85	0.53	1.62	0.25	76.65
	7	2.58	4.63	0.79	2.06	0.32	77.24
	8	1.98	6.04	1.09	2.18	0.32	81.36
	9	1.53	7.54	1.46	2.23	0.36	78.28
	10	2.5	7.75	1.23	3.08	0.25	75.25

**Table 4:** Higher heating value (HHV) of raw sludge and THP-treated solid residues.

Sample Condition	HHV (kJ kg^−1^)
Raw sludge	12,034
150 °C	12,888
180 °C	15,725
210 °C	15,191

**Table 5:** Benchmark comparison between full-scale Nanzih Wastewater Treatment Plant and THP-BEAD (Phase 7).

Parameter	Full-Scale WWTP AD	THP–BEAD (Phase 7)
Reactor type	Conventional AD	BEAD (THP 150 °C)
Effective reactor volume (L)	7,200,000	12
Reactor temperature (°C)	35.3	~30
Hydraulic retention time, HRT (day)	55.5	2.58
Biogas Composition (CH_4_%)	66.9	77.4
CH_4_ production (L $L_{R}^{-1}$·d^−1^)	0.049	0.79
Volumetric VSS loading (kg m^−3^·d^−1^)	0.42	4.68
VSS removal efficiency (%)	39.3	61.97

## Data Availability

The original contributions presented in this study are included in the article. Further inquiries can be directed to the corresponding authors.

## References

[1] Gonzalez A., Hendriks A.T.W.M., van Lier J.B., de Kreuk M. (2018). Pre-treatments to enhance the biodegradability of waste activated sludge: Elucidating the rate limiting step. Biotechnol. Adv..

[2] Das T., Al-Waili I., Balasubramanian V., Appleby G., Kaparaju P., Parthasarathy R., Eshtiaghi N. (2024). Process modelling and techno-economic analysis of anaerobic digestion of sewage sludge integrated with wet oxidation using a gravity pressure vessel and thermal hydrolysis. Sci. Total Environ..

[3] Guo H., Oosterkamp M.J., Tonin F., Hendriks A., Nair R., van Lier J.B., de Kreuk M. (2021). Reconsidering hydrolysis kinetics for anaerobic digestion of waste activated sludge applying cascade reactors with ultra-short residence times. Water Res..

[4] Mainali K., Ferreira Mendes K., Nogueira de Sousa R. (2025). Combined Thermochemical Conversion as a Pretreatment of Manure Waste for Environmental Applications: An Overview. Biochar-Applications in Agriculture and Environment.

[5] Ariunbaatar J., Panico A., Esposito G., Pirozzi F., Lens P.N.L. (2014). Pretreatment methods to enhance anaerobic digestion of organic solid waste. Appl. Energy.

[6] Khanh Nguyen V., Kumar Chaudhary D., Hari Dahal R., Hoang Trinh N., Kim J., Chang S.W., Hong Y., Duc La D., Nguyen X.C., Hao Ngo H. (2021). Review on pretreatment techniques to improve anaerobic digestion of sewage sludge. Fuel.

[7] Mirsoleimani Azizi S.M., Haffiez N., Mostafa A., Hussain A., Abdallah M., Al-Mamun A., Bhatnagar A., Dhar B.R. (2024). Low- and high-temperature thermal hydrolysis pretreatment for anaerobic digestion of sludge: Process evaluation and fate of emerging pollutants. Renew. Sustain. Energy Rev..

[8] Cardova A., Deng Z., Budatala J.M., Appels L., Kouba V., Srb M., Jenicek P. (2026). Thermal hydrolysis on the edge of thermophilic anaerobic digestion: A pilot-scale operation experience. Environ. Sci. Water Res. Technol..

[9] Kakar F.L., El Sayed A., Purohit N., Elbeshbishy E. (2020). Volatile Fatty Acids and Biomethane Recovery from Thickened Waste Activated Sludge: Hydrothermal Pretreatment’s Retention Time Impact. Processes.

[10] Xu D., Han X., Chen H., Yuan R., Wang F., Zhou B. (2020). New insights into impact of thermal hydrolysis pretreatment temperature and time on sewage sludge: Structure and composition of sewage sludge from sewage treatment plant. Environ. Res..

[11] Wu L.-J., Li X.-X., Liu Y.-X., Yang F., Zhou Q., Ren R.-P., Lyu Y.-K. (2021). Optimization of hydrothermal pretreatment conditions for mesophilic and thermophilic anaerobic digestion of high-solid sludge. Bioresour. Technol..

[12] Szkadłubowicz K., Mikusińska J., Pozarlik A., Wilk M. (2025). Hydrothermal Treatment of Sewage Sludge Under Different Process Conditions with a Focus on Energy Properties and Resource Recovery. Energies.

[13] Huang Q., Liu Y., Dhar B.R. (2022). A critical review of microbial electrolysis cells coupled with anaerobic digester for enhanced biomethane recovery from high-strength feedstocks. Crit. Rev. Environ. Sci. Technol..

[14] Thanarasu A., Periyasamy K., Subramanian S. (2022). An integrated anaerobic digestion and microbial electrolysis system for the enhancement of methane production from organic waste: Fundamentals, innovative design and scale-up deliberation. Chemosphere.

[15] Tartakovsky B., Mehta P., Bourque J.S., Guiot S.R. (2011). Electrolysis-enhanced anaerobic digestion of wastewater. Bioresour. Technol..

[16] Colantoni S., Molognoni D., Sánchez-Cueto P., De Soto C., Bosch-Jimenez P., Ghemis R., Borràs E. (2024). Bioelectrochemically-improved anaerobic digestion of fishery processing industrial wastewater. J. Water Process Eng..

[17] Singh V., Tartakovsky B., Örmeci B., Li H., Hussain A. (2025). Combining a solid-state submerged fermenter with bioelectrochemically enhanced anaerobic digestion (BEAD) process for enhanced methane (CH4) production from food waste: Effects of the organic loading rates and applied voltages. J. Environ. Chem. Eng..

[18] Dou Z., Dykstra C.M., Pavlostathis S.G. (2018). Bioelectrochemically assisted anaerobic digestion system for biogas upgrading and enhanced methane production. Sci. Total Environ..

[19] Liu S., Liang D., Wang Y., He W., Feng Y. (2025). Impact of carrier capacitance on Geobacter enrichment and direct interspecies electron transfer under anaerobic conditions. Bioresour. Technol..

[20] Singh V., Hussain A., Ormeci B., Pauzé-Foixet J., Nwanebu E., Li H., Tartakovsky B. (2026). High rate bioelectrochemical anaerobic digester for biomethane production from food waste. Bioengineering.

[21] Cardova A., Jenicek P., Srb M., Sykora P., Rosicky J., Appels L. (2025). Intensification of thermophilic anaerobic digestion of sewage sludge by thermal hydrolysis. Water Sci. Technol..

[22] Westerholm M., Castillo M.d.P., Chan Andersson A., Jahre Nilsen P., Schnürer A. (2019). Effects of thermal hydrolytic pre-treatment on biogas process efficiency and microbial community structure in industrial- and laboratory-scale digesters. Waste Manag..

[23] Smetana G., Grosser A. (2024). The Application of an Upflow Anaerobic Sludge Blanket Reactor in the Treatment of Brewery and Dairy Wastewater: A Critical Review. Energies.

[24] O’Dell J. (1993). Method 410.4, Revision 2.0: The Determination of Chemical Oxygen Demand by Semi-Automated Colorimetry.

[25] Bach Q.-V., Chen W.-H., Lin S.-C., Sheen H.-K., Chang J.-S. (2017). Wet torrefaction of microalga *Chlorella vulgaris* ESP-31 with microwave-assisted heating. Energy Convers. Manag..

[26] Zhou M., Taiwo K., Wang H., Ntihuga J.-N., Angenent L.T., Usack J.G. (2024). Anaerobic digestion of process water from hydrothermal treatment processes: A review of inhibitors and detoxification approaches. Bioresour. Bioprocess..

[27] Zhu K., Liu Q., Dang C., Li A., Zhang L. (2021). Valorization of hydrothermal carbonization products by anaerobic digestion: Inhibitor identification, biomethanization potential and process intensification. Bioresour. Technol..

[28] Periyavaram S.R., Bella K., Uppala L., Reddy P.H.P. (2023). Hydrothermal carbonization of food waste: Process parameters optimization and biomethane potential evaluation of process water. J. Environ. Manag..

[29] Lv N., Cai G., Pan X., Li Y., Wang R., Li J., Li C., Zhu G. (2022). pH and hydraulic retention time regulation for anaerobic fermentation: Focus on volatile fatty acids production/distribution, microbial community succession and interactive correlation. Bioresour. Technol..

[30] Kakar F.L., Tadesse F., Elbeshbishy E. (2022). Comprehensive Review of Hydrothermal Pretreatment Parameters Affecting Fermentation and Anaerobic Digestion of Municipal Sludge. Processes.

[31] Liu X., Wang Q., Tang Y., Pavlostathis S.G. (2021). Hydrothermal pretreatment of sewage sludge for enhanced anaerobic digestion: Resource transformation and energy balance. Chem. Eng. J..

[32] Zhang X., Jiao P., Zhang M., Wu P., Zhang Y., Wang Y., Xu K., Yu J., Ma L. (2023). Impacts of organic loading rate and hydraulic retention time on organics degradation, interspecies interactions and functional traits in thermophilic anaerobic co-digestion of food waste and sewage sludge. Bioresour. Technol..

[33] Musa M.A., Idrus S., Hasfalina C.M., Daud N.N. (2018). Effect of Organic Loading Rate on Anaerobic Digestion Performance of Mesophilic (UASB) Reactor Using Cattle Slaughterhouse Wastewater as Substrate. Int. J. Environ. Res. Public Health.

[34] Yan W., Xu H., Lu D., Zhou Y. (2022). Effects of sludge thermal hydrolysis pretreatment on anaerobic digestion and downstream processes: Mechanism, challenges and solutions. Bioresour. Technol..

[35] Şenol H., Elibol E.A., Bianco F., Race M. (2025). Enhancing methane production from pistachio skin via optimized hydrothermal-alkaline pretreatment and Autoregressive Integrated Moving Average modeling. J. Environ. Manag..

[36] Kim D., Lee K., Park K.Y. (2015). Enhancement of biogas production from anaerobic digestion of waste activated sludge by hydrothermal pre-treatment. Int. Biodeterior. Biodegrad..

[37] Tanguay-Rioux F., Monteil-Rivera F., Ye M., Matteau Lebrun F., Vasquez V., Ngoundjo F., Frigon J.-C., Spreutels L. (2024). Conversion of the solid fraction of food waste separated by a screw press using an integrated hydrothermal carbonization and anaerobic digestion process. Waste Manag..

[38] Sharma I., Rackemann D., Ramirez J., Cronin D.J., Moghaddam L., Beltramini J.N., Te’o J., Li K., Shi C., Doherty W.O.S. (2022). Exploring the potential for biomethane production by the hybrid anaerobic digestion and hydrothermal gasification process: A review. J. Clean. Prod..

[39] Wilson C.A., Novak J.T. (2009). Hydrolysis of macromolecular components of primary and secondary wastewater sludge by thermal hydrolytic pretreatment. Water Res..

[40] Bowman K., Elaiuy M., Fudge G., Rutland H., Gambier W., Hembury T., Jobling-Purser B., Fudge T., Kale I., Kyazze G. (2025). Performance Characteristics of a Pilot-Scale Electromethanogenic Reactor Treating Brewery Wastewater. Energies.

[41] Colantoni S., Santiago Ó., Weiler J.R., Knoll M.T., Lapp C.J., Gescher J., Kerzenmacher S. (2024). Comparative study of bioanodes for microbial electrolysis cells operation in anaerobic digester conditions. J. Environ. Chem. Eng..

[42] Flores-Rodriguez C., Nagendranatha Reddy C., Min B. (2019). Enhanced methane production from acetate intermediate by bioelectrochemical anaerobic digestion at optimal applied voltages. Biomass Bioenergy.

[43] Bougrier C., Delgenès J.P., Carrère H. (2008). Effects of thermal treatments on five different waste activated sludge samples solubilisation, physical properties and anaerobic digestion. Chem. Eng. J..

[44] Scherzinger M., Kaltschmitt M. (2021). Thermal pre-treatment options to enhance anaerobic digestibility—A review. Renew. Sustain. Energy Rev..

[45] Płonka I., Kudlek E., Pieczykolan B. (2025). Municipal Sewage Sludge Disposal in the Republic of Poland. Appl. Sci..

[46] Liang Y., Xu D., Feng P., Hao B., Guo Y., Wang S. (2021). Municipal sewage sludge incineration and its air pollution control. J. Clean. Prod..

[47] Fytili D., Zabaniotou A. (2008). Utilization of sewage sludge in EU application of old and new methods—A review. Renew. Sustain. Energy Rev..

[48] Hidaka T., Nakamura M., Oritate F., Nishimura F. (2022). Comparative anaerobic digestion of sewage sludge at different temperatures with and without heat pre-treatment. Chemosphere.

[49] Liu D., Zhang L., Chen S., Buisman C., Ter Heijne A. (2016). Bioelectrochemical enhancement of methane production in low temperature anaerobic digestion at 10 °C. Water Res..

